# Fear of cancer recurrence and change in hair cortisol concentrations in partners of breast cancer survivors

**DOI:** 10.1007/s11764-024-01631-1

**Published:** 2024-07-02

**Authors:** Alyssa L. Fenech, Emily C. Soriano, Arun Asok, Scott D. Siegel, Michael Morreale, Hannah A. Brownlee, Jean-Philippe Laurenceau

**Affiliations:** 1 Department of Psychological & Brain Sciences, University of Delaware, 108 Wolf Hall, Newark, DE USA; 2 Scripps Whittier Diabetes Institute, San Diego, CA USA; 3 Alien Therapeutics Inc, Philadelphia, PA USA; 4 Helen F. Graham Cancer Center & Research Institute, Christiana Care, Newark, DE USA

**Keywords:** Breast cancer, Fear of cancer recurrence, Hair cortisol, Intimate partners, Change models

## Abstract

**Purpose:**

Partners of breast cancer (BC) survivors report high rates of psychological distress including fear of cancer recurrence (FCR). Research suggests that partners may have poorer physical health outcomes than the general population, but little research has examined the physiological biomarkers by which distress may impact partner health outcomes. The current study examined the associations between FCR and changes in hair cortisol among BC partners.

**Methods:**

Male partners (*N* = 73) of early-stage BC survivors provided hair samples during two visits, one after completion of survivors’ adjuvant treatment (T1) and again 6 months later (T2). Two subscales from the Fear of Cancer Recurrence Inventory and one subscale from the Concerns about Recurrence Scale comprised a latent FCR factor at T1. A latent change score model was used to examine change in cortisol as a function of FCR.

**Results:**

Partners were on average 59.65 years of age (*SD* = 10.53) and non-Hispanic White (83%). Latent FCR at T1 was positively associated (*b* = 0.08, *SE* = 0.03, *p* = .004, standardized *β* = .45) with change in latent hair cortisol from T1 to T2.

**Conclusions:**

Results indicated that greater FCR was associated with increases in hair cortisol in the months following adjuvant treatment. This is one of the first studies to examine the physiological correlates of FCR that may impact health outcomes in BC partners.

**Implications for Cancer Survivors.:**

Findings highlight the need for further research into the relationship between FCR and its physiological consequences. Interventions to address partner FCR are needed and may aid in improving downstream physical health outcomes.

The experience of a breast cancer (BC) diagnosis and its subsequent treatments can result in physical and emotional burdens not only for the BC survivor but also for their intimate partners, who often take on the role of informal caregivers [1]. From diagnosis and throughout treatment, partners may encounter chronic stressors such as physical and psychological strain, changes in intimacy, and high levels of unpredictability [1–3]. Prior findings suggest partners and caregivers may experience significant psychological distress early and throughout the cancer continuum including fear of cancer recurrence, anxiety, and depression [4, 5]. Fear of cancer recurrence (FCR) is defined as the “fear, worry, or concern relating to the possibility that cancer will come back or progress” [6]. FCR is increasingly recognized as a significant survivorship concern, and while the vast majority of research aims to examine the experience of FCR among cancer survivors, far fewer studies consider their partners or informal caregivers [5]. Notably, partners often report rates of FCR that are equal to or exceeding the rates of survivors with an estimated 48% of partners reporting clinically significant levels of FCR [5, 7, 8]. Research suggests that without intervention, elevated FCR remains stable and chronic over time and is associated with disruptions in emotional and physical functioning and reductions in quality of life [9]. Chronic psychological distress associated with the BC experience, including FCR, may increase a partner’s risk for physical health complications, such as cardiovascular disease, increased blood pressure, fatigue, or poor immune function [10–13].

Biomarkers of neuroendocrine function provide insight into the psychophysiological relationship between stress and physical health. During periods of acute stress, the hypothalamic–pituitary–adrenal (HPA) axis is activated and releases hormones, which in turn stimulates the adrenal glands to release the glucocorticoid cortisol [14]. Given its stress-related modulation, cortisol is often used as a biological marker of acute and chronic stress reactivity. Importantly, HPA-axis activation and the release of cortisol permits the temporary inhibition of immune and inflammatory responses, thereby facilitating the body’s overall physiological response to perceived threat [14, 15]. Over time, cortisol reaches a threshold and triggers the deactivation of the HPA axis to ultimately inhibit the stress response [14, 15]. Notably, prolonged stress can have a deleterious effect by producing a chronic and sustained activation of the HPA axis resulting in downstream health complications such as cardiovascular disease, diabetes, and poor immune function [16–19].

A growing body of research suggests that the physiological correlates of chronic psychological stress may mediate the longer-term physical health outcomes of partners of cancer survivors [20]. Indeed, a number of studies have found that changes in HPA-axis function are associated with several chronic psychological concerns such as post-traumatic stress, depression, anxiety, and marital strain among cancer survivors and partners [18, 20–23]. These studies highlight a more nuanced, but poorly understood, role for HPA function and its major hormone, cortisol, in chronic physical and mental-health-related disorders. Cortisol fluctuates during times of chronic stress and is influenced by features of the stressor such as the time since the stressor onset, controllability of the stressor, nature of the threat, and stressor-associated emotions elicited over time [24]. While there is limited research on the physiological correlates of FCR, prior research has assessed cortisol in relation to anxiety, which shares a mechanistic overlap with FCR [25]. In particular, generalized anxiety disorder, like FCR, is characterized by chronic excessive anxiety and worry and is associated with elevated cortisol compared to non-anxious controls [26].

Cortisol is traditionally captured from plasma, saliva, or urine samples in order to examine how levels change in response to an acute stressor or diurnally fluctuate over the proceeding 12–24 h [27]. These collection methods can present logistical barriers for participants (e.g., daily measurements, storage, and blood draws) with a potential confound of state variability arising from preexisting medical conditions or health behaviors such as circadian variation [27–29]. By contrast, the assessment of cortisol from hair samples has emerged as a valid and reliable method for capturing aggregate cortisol output over the prior 2–4 months, therefore providing a retrospective assessment of cumulative stress [28, 30]. Relative to other collection methods, cortisol measured in hair samples allows for a longer-term assessment of cortisol output during periods when a chronic stressor is most salient (e.g., cancer diagnosis and treatment). Thus, not only does hair cortisol provide an aggregate output of cortisol over time, but it also has the added benefit of a lower burden to the participant.

Research into the physiological mechanisms by which chronic stress may relate to poorer physical health outcomes in partners of cancer survivors is limited. FCR is a top psychosocial concern for survivors and partners that may develop early in the cancer trajectory and be stable into survivorship, yet no research to date has examined the physiological correlates of FCR. The current observational study sought to examine the relationship between FCR and cortisol output, particularly examining if FCR was associated with change in hair cortisol concentrations among partners after the completion of adjuvant treatment for the BC survivor. It was hypothesized that greater levels of FCR near the end of adjuvant treatment would be positively associated with changes in cortisol in the months following treatment as the couple transitions into survivorship.

## Method

### Participants and procedures

The current study is a secondary analysis of data collected as part of a larger longitudinal study conducted at the Christiana Care Health System aimed at examining the experience of couples coping with cancer. The parent study was approved by the Christiana Care Health System and the University of Delaware IRB (FWA00006557; CCC# 33,026). Early-stage BC survivors and their partners were recruited from Christiana Care Health System from 2013 to 2015. Couples were eligible to participate if they were over the age of 18, able to read and speak English, currently cohabitating, living within an hour of the cancer center, if the survivor was diagnosed with early-stage BC (stage 0-IIIA), received recent surgery for treatment of their disease, and had no prior cancer diagnoses. All participants who enrolled in the study provided written informed consent prior to performing study procedures.

Participants enrolled in the study shortly after the survivor’s BC surgery and were followed to the 1-year post-treatment mammogram. Participants were compensated for their time and effort in the study. Supplementary information on the parent study procedures can be found in prior publications [31, 32]. The current study included two timepoints from the parent study: the first timepoint (T1) was shortly after the end of adjuvant treatment, and the second timepoint (T2) was approximately 6 months post-treatment. Shortly after the survivor completed adjuvant chemotherapy or radiation, survivors and partners completed a post-treatment self-report questionnaire including measures of FCR via electronic survey, independently. This timepoint was of particular interest for the current study to explore if FCR is salient as couples transition out of active treatment with less regular medical team contact and into long-term survivorship. At this first timepoint (T1) and 6 months after treatment completion (T2), hair samples were collected from partners to assess change in cortisol.

### Hair cortisol procedures

Hair samples were collected from partners by a research assistant during two home visits. After completion of the aforementioned post-treatment questionnaire, couples were contacted to schedule the first home visit. The first home visit took place an average of 20 days (*SD* = 14.5) after the post-treatment (T1) questionnaire, which was an average of 5.4 months (*SD* = 2.1) after the survivor’s initial BC surgery. The second home visit took place approximately 2 weeks before the survivor’s annual follow-up mammogram, which was on average, 5.7 months (*SD* = 2.5) after the first home visit. Research assistants aimed to collect approximately 3–4 cm of hair with 1 cm corresponding to 1 month of cortisol output [33, 34]. Partners’ hair was cut as close to the scalp as possible, and the length of each sample was recorded at the time of collection. The collected hair was wrapped together and placed in aluminum foil, and the portion closest to the scalp was marked when necessary. The aluminum foil samples were then stored at room temperature to avoid any potential issues with analysis from repeated freeze/thaw cycles [34]. Across time points, the average extracted hair length was 4.3 cm (*SD* = 4.1).

Cortisol was extracted from hair using procedures similar to the in-depth procedures outlined by Meyer and colleagues [30]. Approximately 30.98 mg (*SD* = 24.01) of hair for each participant was transferred to a Triple-Pure 2 mL micro-centrifuge tube containing 6 mm high-impact zirconium beads (Stellar Scientific, Baltimore, MD). Samples were sealed and homogenized on a Bead Blaster 24 Homogenizer (Benchmark Scientific, Sayreville, NJ) at 6.5 speed for 10 cycles, 30 s per cycle, and a 10 s delay between cycles. Tubes were briefly (approx. 20 s) centrifuged and homogenized again for 5 cycles. Following homogenization, samples were centrifuged (4000 rpm for 4 min.), and 1.5 mL of molecular biology-grade methanol was added to each tube. Samples were rotated at room temperature for 24 h. After methanol incubation, tubes were centrifuged for 8 min. at 7000 rpm. One milliliter of methanol was recovered and transferred to a sterile 1.5 mL microcentrifuge tube. Methanol was evaporated using a sample concentrator for ~ 3 h at 35 °C. The dried sample was then reconstituted in 200 µL hair cortisol ELISA assay buffer, gently vortexed, and stored at − 20 °C until ELISA analysis.

## Measures

### Hair cortisol assay and calculation

Assays were performed using the Salimetrics, Inc. High-Sensitivity Cortisol Enzyme Immunoassay Kit according to the manufacturer’s instructions. Hair was collected at two timepoints, and both timepoints were run in duplicate on the same assay plate to reduce measurement variability across plates. Additionally, a collection of samples was pooled and frozen as a control. Control samples were included on each assay plate. Any sample that fell outside of the range of 0.007 μg/dl ≤  ×  ≤ 3.417 μg/dl was re-assayed to exclude the possibility of experimenter error and then excluded from analysis according to the guidelines provided by the manufacturer (Salimetrics Inc.). Intra- and inter-assay coefficients of variation were 2.74% and 6.79%, respectively. The final amount of cortisol present in each participant’s hair was calculated from the µg/dl value obtained from the salivary cortisol kit [30]. Hair cortisol was represented in pg/mg using the following transformation: hair cortisol pg/mg = (µg/dl cortisol output ÷ mg of hair) × (mL methanol added ÷ mL methanol recovered) × mL sample assay buffer × 10,000. In line with prior literature, cortisol was log-transformed to handle skewness in current analyses [35, 36].

### Fear of cancer recurrence

A latent FCR factor was estimated utilizing three subscales from two validated measures tapping into core aspects of FCR. Measures administered to partners were reworded to assess their fears related to the possibility of the survivor’s BC recurring or progressing. The Fear of Cancer Recurrence Inventory (FCRI) is a 42-item measure including seven subscales [37]. The *severity subscale* is nine items assessing intrusive thoughts about perceived risk of recurrence and the *distress subscale* is four items assessing emotional responses to thoughts of recurrence. Responses were reported on a Likert scale ranging from 0 (*not at all*) to 4 (*all the time*), with higher scores indicating greater FCR. The Concerns about Recurrence Scale (CARS) is a 30-item scale with five subscales examining various emotional and functional domains related to FCR [38]. The *overall subscale* includes four items assessing emotional distress associated with FCR and intensity of FCR with response items ranging from 1 (*not at all*) to 6 (*all the time/extremely*), again with higher scores indicating greater FCR. These subscales were selected as they capture the general construct of FCR and its emotional and cognitive components, while other subscales on the FCRI and CARS assess antecedents, consequences, or content of recurrence fears. In the present study, the severity (*α* = 0.82), distress (*α* = 0.91), and CARS overall (*α* = 0.90) subscales demonstrated strong reliability and the three indicators loading on a single latent variable had strong internal consistency (*ω* = 0.90).

### Statistical analyses

**Fig. 1:**
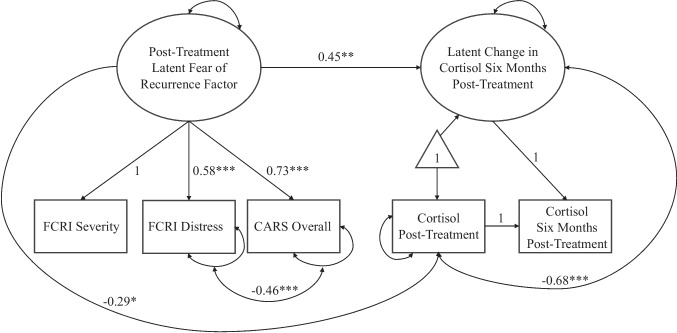
Path model with standardized parameter estimates. *Note.* FCRI, Fear of Cancer Recurrence Inventory; *CARS*, Concerns about Recurrence Scale. Covariates were omitted for visual clarity. ***p < .001, **p < .01, *p < .05

Data were analyzed using structural equation modeling in *Mplus Version 8* [39]. Two-tailed significance tests were conducted using an alpha of 0.05. Descriptive statistics were computed, and variable distributions were examined to verify model assumptions were met. A latent change score approach was used to model a latent estimate of change in cortisol (log-transformed) from the end of adjuvant treatment to 6 months post-treatment (see right side of path model in Fig. 1). This approach makes use of all available data, including participants who only contributed data at one timepoint under the assumption of missing at random [40]. In the same model, an error-free latent FCR factor was estimated utilizing the scores of the FCRI severity, FCRI distress, and CARS overall subscales scaled to the metric of the FCRI subscales (range = 0–36). The cortisol latent change score was then regressed on the latent FCR factor to test the study hypothesis that greater FCR near the end of adjuvant treatment would be associated with increases in hair cortisol in the following 6 months. To control for individual differences in the chronicity of exposure, partner hair length at the first and second timepoints was grand mean centered and included as covariates in the model. To consider alternative biobehavioral factors as potential confounders, partner BMI and age were included as covariates. The latent FCR factor and post-treatment cortisol observed variable were allowed to covary since they were assessed concurrently. The full estimated path model is depicted in Fig. 1. Model fit was examined using model chi-square, comparative fit index (CFI), root mean square error of approximation (RMSEA), and standardized root mean square residual (SRMR) indices.

## Results

The total sample of the parent study consisted of 79 partners; of these, 4 were excluded due to completely missing data, and 2 female sex assigned at birth partners were removed due to observed sex differences in variables of interest [29, 41]. Therefore, this led to a final sample of 73 partners included in the present study. Sociodemographic characteristics of partners and medical characteristics of survivors are shown in Table 1. Partners were all of male sex and, on average, 59.65 (*SD* = 10.53) years of age, White (83%; none were Hispanic/Latino), and married (95%). Thirteen percent of partners were identified as Black or African-American, with the remaining 2% identifying as Asian. Nearly 42% of participants reported an annual family income greater than $100,000, and 52% of partners were employed full-time. The majority of survivors were diagnosed with stage I BC (49%), followed by 35% with stage II, 13% with stage 0, and 1% with stage IIIA. Survivors reported receiving hormonal therapy (76%), radiation treatment (71%), and chemotherapy (34%) for treatment of their BC.

**Table 1:** Baseline participant characteristics (*N* = 73)

	No. of mean	% or SD
Partner age at consent (years)	59.65	10.53
Partner sex		
Male	73	100%
Partner race		
White	61	83.6%
Black	10	13.7%
Asian	2	2.7%
Non-Hispanic/Latino	73	100%
Relationship status		
Married	70	95.9%
Engaged	1	1.4%
Committed relationship	2	2.7%
Annual household income		
< $60 k	14	19.2%
$60 k–99 k	27	37%
> $100 k	31	42.5%
Partner employment		
Full-time	38	52.1%
Part-time	9	12.3%
Not employed	26	35.6%
Survivor BC stage		
Stage 0	10	13.7%
Stage I	36	49.3%
Stage II	26	35.7%
Stage IIIA	1	1.4%
Survivor BC treatment		
Radiation	52	71.2%
Chemotherapy	25	34.2%
Hormonal therapy	56	76.7%

Descriptive statistics and bivariate correlations for study variables are shown in Table 2. Model fit was initially deemed adequate when including the full sample of partners: *χ*^2^ (8) = 18.96, *p* = 0.015; RMSEA = 0.135 (90% CI: 0.056, 0.215); CFI = 0.922; *SRMR* = 0.065. After the removal of the two female partners, the addition of BMI and age as covariates, and allowing for correlated residuals between two observed indicators, model fit was deemed acceptable: *χ*^2^ (11) = 14.46, *p* = 0.21; RMSEA = 0.06 (90% CI: 0.00, 0.14); CFI = 0.97; SRMR = 0.06. The correlated residuals between the FCRI distress and CARS overall subscales were informed by post-hoc modifications, consistent with the notion that these two subscales highly overlap in construct, and both capture the more emotional components of FCR [37, 38, 42]. Although an RMSEA < 0.06 is typically preferred, it is not considered to be a reliable model fit indicator in models with few degrees of freedom and small sample sizes, as in the present study [43]. Key standardized parameter estimates are shown in Fig. 1. The standardized loadings of FCRI severity, FCRI distress, and CARS overall were 1, 0.58, and 0.73 (all *p* < 0.001).

**Table 2:** Bivariate correlations, means, and standard deviations of key study variables

	1	2	3	4	5
1. Post-treatment cortisol (T1)	(1)				
2. Six-month cortisol (T2)	0.19	(1)			
3. FCRI severity	− 0.25	0.16	(1)		
4. FCRI distress	− 0.27	0.12	0.58**	(1)	
5. CARS overall	− 0.09	0.20	0.73**	0.68**	(1)
Means	0.13	0.19	6.68	1.64	10.42
SDs	0.61	0.48	4.45	2.57	4.74
*N*	50	45	72	72	72

*Note. FCRI*, Fear of Cancer Recurrence Inventory; *CARS*, Concerns about Recurrence Scale. FCR (variables 3–5) was measured post-treatment. Cortisol was log-transformed

^*^p < 0.05 level (2-tailed)

^**^p < 0.01 level (2-tailed)

In the final latent change score model, latent FCR near the end of adjuvant treatment (T1) was significantly associated with change in cortisol from T1 to T2 (*b* = 0.08, *SE* = 0.03, *p* = 0.004, standardized *β* = 0.45), indicating that a one-unit increase in FCR was associated with a 0.08-unit increase in hair cortisol from the end of adjuvant treatment to 6 months post-treatment. Neither hair length at timepoint one (*b* =  − 0.02) nor two (*b* =  − 0.03) was a statistically significant predictor of change in cortisol (both *p* > 0.4). Among biobehavioral factors, neither partner age (*b* =  − 0.01) nor BMI (* b* = 0.01) were significant covariates (both *p* > 0.2). The latent change score model yielded an *R*^2^ of 0.27, indicating that 27% of the variance in hair cortisol change was explained by the final FCR model including covariates. The mean latent change in hair cortisol was estimated to be 0.13, indicating a small but non-significant increase in cortisol over time (*p* = 0.24). Nevertheless, there were significant individual differences in latent change in cortisol (variance = 0.40, *SE* = 0.09, *p* < 0.001) suggesting heterogeneity remaining in the observed change.

## Discussion

The current study sought to investigate the relationship between FCR and HPA function through the examination of change in hair cortisol output in a sample of partners of BC survivors. Results supported the study hypothesis in that greater FCR among partners near the end of adjuvant treatment was associated with increases in hair cortisol concentrations in the following 6 months post-treatment. Specifically, we found a moderate-sized effect of partner FCR on change in hair cortisol, where a one standard deviation unit increase in FCR was associated with nearly half a standard deviation unit increase in cortisol over the following 6 months post-treatment. Study findings highlight the burden of FCR among partners of cancer survivors as well as the potential physiological correlates.

Results concur with existing literature on chronic stress and HPA function and provide novel insights into the relationship between FCR and long-term cortisol output among partners of BC survivors. A number of prior studies have reported on the associations between chronic stress and HPA activity among partners and informal caregivers of patients with cancer, yet the integration of biomarkers into the partner experience is a rather new and burgeoning area of research [20]. In a scoping review of cancer caregiver biomarkers, Park and colleagues reported salivary or serum cortisol to be the most commonly used marker of neuroendocrine function; however, findings are generally inconsistent across studies with variability in significance between caregivers and controls and in directionality of results [20]. Only one prior study has utilized hair cortisol methods in a cancer caregiver sample in which the study objective was to assess differences in chronic stress between geriatric and cancer caregivers [44]. Results of this small cross-sectional study reported that hair cortisol concentrations were not significantly associated with measures of perceived stress [44]. Additionally, a study on spousal caregivers of patients with dementia utilized hair cortisol methodology and reported significantly elevated levels of cortisol that were positively associated with greater depressive symptoms among spousal caregivers compared to matched non-caregiver controls [45]. The results of the present study add to the growing literature on HPA function in partners and informal caregivers of those living with chronic illness. Findings provide preliminary evidence for the relationship between FCR and physiological processes among BC partners, yet further longitudinal research is warranted with consistent biomarker methodology to assess the consequences of cancer-specific psychological distress faced by partners of cancer survivors.

In the current study, 27% of the variance in cortisol change was explained by the latent FCR model, highlighting the need for further research to examine the multifaceted factors of FCR and cortisol. The present study only assessed the emotional and cognitive components of FCR at one timepoint, excluding antecedents (e.g., triggers) and consequences (e.g., coping style) as well as change in FCR over time. FCR triggers such as survivors’ physical symptoms or medical appointments, as well as the importance of worry, are considered essential factors in the development and maintenance of FCR and may be significant features of cumulative cortisol output [8, 51]. Further, during periods of chronic stress, including a cancer diagnosis and treatment, couples face individual and collective stress and may engage in dyadic and individual coping that can help or hinder overall psychosocial adjustment [46, 47]. Specifically, in couples coping with cancer, approach-oriented coping is associated with greater cancer-specific adjustment, while avoidant-oriented coping is associated with a number of negative outcomes, such as reduced marital satisfaction [46, 47]. Notably, prior research reports that greater use of avoidant-oriented coping is associated with increased FCR during the first year after a BC diagnosis [48]. The current study lacked a measure of coping thus excluding important information on how couples cope with chronic stress, which may be a significant factor in the development and maintenance of FCR as well as cortisol output over time. Although FCR is reported as a stable construct, it may fluctuate with associated antecedents and consequences resulting in change over time; therefore, future research should assess multiple domains of FCR longitudinally to explore change in relation to cumulative cortisol output throughout survivorship. Additionally, chronic psychological stress has the ability to increase or decrease cortisol output, with a person’s appraisal of the stressor such as perceived control and chronicity greatly relating to physiological function [24, 49]. Research suggests illness perceptions such as controllability and threat appraisals such as perceived consequences or risk of disease may be significant factors associated with FCR [50]. Future research should extend these findings with a larger partner sample to explore additional domains of stress, coping, and change in FCR in relation to cumulative cortisol output.

Partners in the current sample reported mild-to-moderate levels of FCR across the three subscales near the end of adjuvant treatment. Notably, the majority of partner FCR research, including the current study, rely on patient measures that are not validated in partner or caregiver samples, thus limiting our understanding of clinically significant thresholds and the magnitude of scores [5, 8]. Nevertheless, prior research suggests a reciprocal relationship of FCR between partners and survivors; therefore, understanding the dyadic relationship of FCR may aid in our understanding of partner FCR and its consequences as well as inform FCR intervention development to target both members of the dyad [31, 52]. Psychosocial interventions, specifically cognitive behavioral and mind–body programs, have demonstrated significant reductions in FCR and cortisol among cancer survivors [53, 54]. Additionally, a recent couple-focused skill training intervention based on cognitive-behavioral theory found significant reductions in FCR and increases in hope and communication among BC survivors and their partners in the intervention group [55]. Future dyadic interventions should consider cognitive-behavioral and mind–body programs in a dyadic framework as these mechanisms may aid in reducing the burden of FCR among partners and its potential physiological consequences.

## Limitations

The current study had several limitations to note. The sample was limited in size and was highly homogenous in terms of sociodemographic and medical characteristics and recruited from a single community cancer center. The sample may limit the generalizability of these results to partners of different sociodemographic backgrounds, relationship types, regions, or partners of patients with advanced stage BC. Further, the limited sample size may have impacted our model fit and power in detecting effects. The study utilized the novel methodology of hair sample collection for cortisol extraction; however, the study was limited on variables that may affect cortisol concentrations in hair such as hair treatments, personal hygiene, medications, or health behaviors as well as medical illnesses that affect cortisol output such as Cushing syndrome or Addison’s disease [28, 56]. Furthermore, the analyses examined the relationship between FCR and cortisol change, with only one timepoint measuring FCR and two assessing cortisol. Future research with larger sample sizes should investigate FCR and cortisol longitudinally with more repeated assessments to further examine the relationship over time and to reduce potential threats to internal validity (e.g., regression to the mean). The measurement of FCR utilized a latent variable approach allowing for the inclusion of three FCR subscales. However, the FCR measures were validated in patient samples and were reworded to be used with partners in the parent study. The rewording of patient measures for partner or caregiver samples is a common but alarming practice in caregiver FCR research [5]. Without proper partner or caregiver, FCR measures, or evidence of measurement invariance, we may be excluding significant role stress, worries, or fears that are unique to the partner perspective [5]. Future research is needed to develop and/or test partner and caregiver measures to accurately assess the experience of partner FCR and its associated antecedents and consequences.

## Conclusions

FCR is an enduring survivorship concern for cancer survivors and their partners. The present study examined the relationship between FCR and change in hair cortisol output among partners of BC survivors in the first year after diagnosis and treatment. Results found that FCR near the end of adjuvant treatment was associated with increases in hair cortisol output in the following 6 months post-treatment. This preliminary evidence requires further replication in a larger longitudinal study to elucidate the physiological consequences of FCR. Results highlight the need for interventions to address partner FCR which may aid in mitigating downstream physical health outcomes.

## Data Availability

The data that support the findings of this study are available from the corresponding author upon request.
